# Sample Size Estimates for Cluster-Randomized Trials in Hospital Infection Control and Antimicrobial Stewardship

**DOI:** 10.1001/jamanetworkopen.2019.12644

**Published:** 2019-10-04

**Authors:** Natalia Blanco, Anthony D. Harris, Laurence S. Magder, John A. Jernigan, Sujan C. Reddy, Justin O’Hagan, Kelly M. Hatfield, Lisa Pineles, Eli Perencevich, Lyndsay M. O’Hara

**Affiliations:** 1 Department of Epidemiology and Public Health, University of Maryland School of Medicine, Baltimore; 2 Division of Healthcare Quality Promotion, Centers for Disease Control and Prevention, Atlanta, Georgia; 3 Department of Internal Medicine, Carver College of Medicine, University of Iowa, Iowa City

## Abstract

**Importance:**

An important step in designing, executing, and evaluating cluster-randomized trials (CRTs) is understanding the correlation and thus nonindependence that exists among individuals in a cluster. In hospital epidemiology, there is a shortage of CRTs that have published their intraclass correlation coefficient or coefficient of variation (CV), making prospective sample size calculations difficult for investigators.

**Objectives:**

To estimate the number of hospitals needed to power parallel CRTs of interventions to reduce health care–associated infection outcomes and to demonstrate how different parameters such as CV and expected effect size are associated with the sample size estimates in practice.

**Design, Setting, and Participants:**

This longitudinal cohort study estimated parameters for sample size calculations using national rates developed by the Centers for Disease Control and Prevention for methicillin-resistant *Staphylococcus aureus* (MRSA) bacteremia, central-line–associated bloodstream infections (CLABSI), catheter-associated urinary tract infections (CAUTI), and *Clostridium difficile* infections (CDI) from 2016. For MRSA and vancomycin-resistant enterococci (VRE) acquisition, outcomes were estimated using data from 2012 from the Benefits of Universal Glove and Gown study. Data were collected from June 2017 through September 2018 and analyzed from September 2018 through January 2019.

**Main Outcomes and Measures:**

Calculated number of clusters needed for adequate power to detect an intervention effect using a 2-group parallel CRT.

**Results:**

To study an intervention with a 30% decrease in daily rates, 73 total clusters were needed (37 in the intervention group and 36 in the control group) for MRSA bacteremia, 82 for CAUTI, 60 for CLABSI, and 31 for CDI. If a 10% decrease in rates was expected, 768 clusters were needed for MRSA bacteremia, 875 for CAUTI, 631 for CLABSI, and 329 for CDI. For MRSA or VRE acquisition, 50 or 40 total clusters, respectively, were required to observe a 30% decrease, whereas 540 or 426 clusters, respectively, were required to detect a 10% decrease.

**Conclusions and Relevance:**

This study suggests that large sample sizes are needed to appropriately power parallel CRTs targeting infection prevention outcomes. Sample sizes are most associated with expected effect size and CV of hospital rates.

## Key Points

**Question:**

What are the estimated sample sizes needed to adequately power parallel cluster-randomized trials with common health care–associated infection outcomes, and how do the parameters affect these estimates in the field of hospital infection control and antimicrobial stewardship?

**Findings:**

This cohort study found that large sample sizes were needed to appropriately power cluster-randomized trials in the field of hospital epidemiology, because the outcomes are rare. The expected effectiveness of the intervention and the strength of correlation within a cluster had the greatest association with the estimated sample size.

**Meaning:**

These findings suggest that better-designed cluster-randomized trials in the field of hospital epidemiology and antimicrobial stewardship that appropriately account for clustering and realistic effect sizes will provide a more reliable evidence base for advancing recommendations and best practices.

## Teaser

This study of national rates of health care–associated infections estimates the number of hospitals needed to power parallel cluster-randomized trials of interventions and evaluates how different parameters such as coefficient of variation and expected effect size are associated with the sample size estimates in practice.

## Introduction

Hospital or health care epidemiology is the branch of epidemiology that focuses on the understanding, prevention, and control of health care–associated infections (HAIs), which are acquired in a health care setting.^1^ On any given day, approximately 1 in 25 hospital patients has at least 1 HAI.^2^ In the era of ongoing emergence of multidrug-resistant organisms, evaluating the effectiveness of infection control and antibiotic stewardship interventions has become a critical domain of research globally.

In the field of hospital epidemiology, the cluster-randomized trial (CRT) design is frequently used. In this study design, intact social units or clusters of individuals such as wards, intensive care units (ICUs), or hospitals rather than independent individuals are randomized to intervention groups.^3,4^ This is often the design of choice because randomization cannot occur at the individual patient level owing to ethical issues or group-level confounding variables, also known as *treatment group contamination*.^4,5^ Furthermore, in practice, this design has several advantages compared with individually randomized trials, such as increased administrative efficiency, reduced risk of treatment group contamination, and likely improvement of participant compliance.^4^

An important step in designing, executing, and evaluating CRTs is understanding the correlation and thus nonindependence that exists among individuals in a cluster. For example, in CRTs in which the ICU is the unit of randomization, such as the Benefits of Universal Glove and Gown (BUGG) study or the Randomized Evaluation of Decolonization vs Universal Clearance to Eliminate MRSA (REDUCE MRSA) trial,^6,7^ patients are not independent within the same ICU; factors that affect acquisition and infection with antibiotic-resistant bacteria are correlated within a cluster (eg, cluster antibiotic-resistant colonization pressure, cluster hand hygiene compliance, cluster severity of illness). Intraclass correlation is the lack of independence among individual patients within the same cluster.^4^ The intraclass correlation coefficient (ICC) is mathematically defined as the ratio of the between-cluster variance to the total variance (between-cluster and within-cluster variance).^8,9^ Thus, the ICC increases when the between-cluster variation increases, but the ICC decreases when the within-cluster variation increases. This lack of independence within clusters creates special methodological challenges, particularly a reduced statistical efficiency.^4^ Therefore, accounting for clustering in power calculations a priori and in the final statistical analysis is critically important.

A related parameter that is often used in sample size calculations is the coefficient of variation (CV) of the cluster-specific outcome rates. The CV is defined as the SD of the hospital-specific outcome rates divided by the overall mean outcome rate. The higher the intraclass correlation, the higher the CV, leading to larger required sample sizes for CRTs than for individual patient-level randomized trials.

In hospital epidemiology, there is a shortage of CRTs that have published their ICC or CV, making prospective sample size calculations difficult for investigators. Furthermore, the lack of methodological rigor when conducting and/or reporting power calculations in hospital epidemiology CRTs has led to a number of potentially underpowered studies.^10,11,12,13^ This issue has also been described in other fields in epidemiology.^14^ If we hope to abate the multidrug-resistant organism crisis internationally, we need evidence-based interventions guided by well-designed, adequately powered CRTs or quasi-nonrandomized trials.

The aim of our study was to estimate the sample sizes needed to adequately power parallel CRTs with HAI or colonization and acquisition of antibiotic-resistant bacteria as outcomes. In addition, we aimed to demonstrate how different parameters such as CV and expected effect size are associated with the sample size estimates in practice. This information should provide future researchers with valuable data and should provide hospital epidemiologists with a better understanding of the potential study sizes needed to answer important hospital epidemiology research questions and ultimately to inform policy and funding allocation.

## Methods

### Data Sources

Data were collected for this study from June 2017 through September 2018. Because this study did not include human participants, the institutional review board of the University of Maryland School of Medicine, Baltimore, waived the need for approval and informed consent. This study followed the Strengthening the Reporting of Observational Studies in Epidemiology (STROBE) reporting guidelines.

To obtain estimates of the rate of hospital-onset events for central-line–associated bloodstream infections (CLABSI), methicillin-resistant *Staphylococcus aureus* (MRSA) bacteremia, catheter-associated urinary tract infections (CAUTI), and *Clostridium difficile* infections (CDI), we used measures developed by the Centers for Disease Control and Prevention, collected by the National Healthcare Safety Network, and reported by the Centers for Medicare & Medicaid Services Hospital Compare program.^15^ Measures of HAI were publicly reported in the Hospital Compare program for 3931 acute care hospitals in 2016; CLABSI and CAUTI data were reported for ICUs and select wards; and MRSA bacteremia and CDI data were reported facility-wide from laboratory-identified events. The median number of ICU beds for these hospitals was 12, and 46% reported having a medical school affiliation.^16^ In addition, we calculated ICU rates of acquisition of MRSA and vancomycin-resistant enterococci (VRE) based on the baseline period data in 2012 of the BUGG study.^5^

### Statistical Analysis

Data were analyzed from September 2018 through January 2019. For this study, infection parameters were calculated based on data from all acute care hospitals across the United States participating in the Hospital Compare program that reported at least 1 infection for the outcome of interest. Rates of infections were calculated using the number of observed HAI cases and patient- or device-days from 2016 for each hospital. The mean cluster-specific rate for each infection or acquisition was calculated by taking a weighted mean of the cluster-specific rates providing greater weights to the larger sites (Table).

**Table.** Parameter Estimates for Health Care–Associated Infection and MRSA and VRE Acquisition Rates

Outcomes^a^	No. of Observed Cases	No. of Patient-Days or Device-Days^b^	No. of Hospitals	Mean Cluster-Specific Rate per Patient-Day or Device-Day	SD of Hospital-Specific Acquisition Rates per Patient-Day or Device-Day	Estimated CV of Hospital-Specific Acquisition Rates (SE)^c^	Mean Cluster Size/d^d^
MRSA bacteremia	7857	134 687 225	1682	0.000055	0.00003	0.5520 (0.0176)	219.4
CAUTI	20 371	19 500 649	2293	0.000955	0.00066	0.6968 (0.0147)	23.3
CLABSI	15 974	18 531 845	2029	0.000815	0.00044	0.5507 (0.0136)	25.0
CDI	92 886	138 491 904	3055	0.000616	0.00027	0.4417 (0.0058)	124.2
Acquisition							
MRSA	136	17 023	20	0.008320	0.00486	0.5840 (0.2806)	7.1
VRE	230	16 510	20	0.014260	0.00738	0.5180 (0.2183)	6.9

Abbreviations: CAUTI, catheter-associated urinary tract infections; CDI, *Clostridium difficile* infection; CLABSI, central-line–associated bloodstream infections; CV, coefficient of variation; MRSA, methicillin-resistant *Staphylococcus aureus*; VRE, vancomycin-resistant enterococci.

^a^ The MRSA bacteremia, CAUTI, CLABSI, and CDI estimates are derived from 2016 Hospital Compare data; MRSA and VRE acquisition estimates, from the Benefits of Universal Glove and Gown Study (BUGG) study.^6^

^b^ Patient-days and device-days are as reported to the National Healthcare Safety Network.

^c^ The CVs were calculated by dividing the SD of hospital-specific acquisition rates by the mean rate. The SEs were estimated based on 1000 bootstrap samples.

^d^ Defined as the number of patient- or device-days per cluster per day. This was calculated from the Hospital Compare or BUGG data by dividing the total number of patient- or device-days by the total number of hospitals and total study days.

Sample size calculations for CRTs also depend on the variation between clusters with respect to the rate of the outcomes. We used hospital-specific National Healthcare Safety Network data and ICU-specific rates during the baseline period of the BUGG study to calculate estimates of the SD of the mean daily rates for each outcome. This estimate was based on the degree to which the variation between clusters in observed rates exceeded what would be expected had they all had the same underlying rate. Details are in the equation in the eMethods in the Supplement. The CV was calculated by dividing the SD by the overall daily rate. Finally, the standard errors of the CV estimates were calculated using 1000 bootstrap samples, each based on sampling hospitals with replacement (Table).

To estimate the total sample sizes (number of clusters overall) or the number of clusters needed in the intervention group and in the control group, we used R package CRTSize, version 1.0 (R Project for Statistical Computing), and the n4incidence function.^17,18,19^ Parameters included in the function were as follows: (1) the anticipated daily incidence rate in the experimental group with the outcome; (2) the anticipated daily incidence rate in the control group with the outcome; (3) the anticipated mean cluster size (ie, number of patient-days or device-days per cluster); (4) the planned follow-up time for the study (days); (5) the CV of cluster-specific rates; (6) the allocation ratio (ratio of patients in the intervention group to the control group); (7) the desired α or type I error rate; and (8) the desired level of power or type II error rate.

To study the association of varying effect size with sample size requirements, the estimated intervention effect sizes were set at 10%, 30%, and 50% based on commonly reported ranges of the effect of interventions on HAI outcomes. To evaluate the association of the CV estimate with sample size, this parameter was estimated from the data and was then increased and decreased by 0.1 with a type II error rate of 0.20 or 0.10 (ie, power of 0.80 or 0.90).

## Results

**Figure 1.**
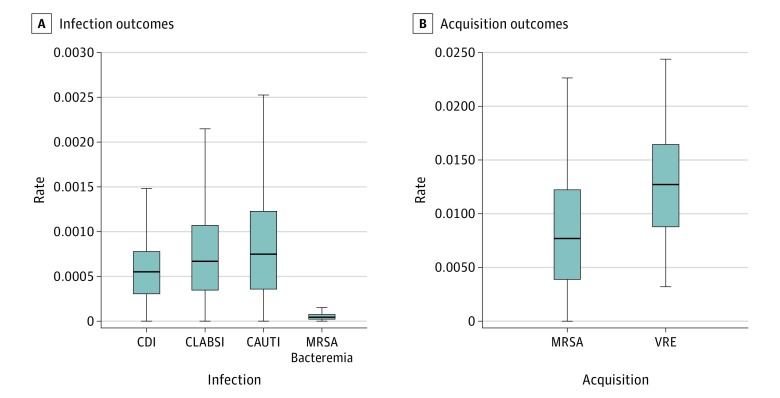
Cluster-Specific Rates Variation by Outcome Rates are per patient-day or device-day, depending on the outcome. All box plots exclude outside values. Boxes indicate interquartile range; horizontal line, median; and upper and lower whiskers, variability outside the upper and lower quartiles. CAUTI indicates catheter-associated urinary tract infections; CDI, *Clostridium difficile* infection; CLABSI, central-line–associated bloodstream infections; MRSA, methicillin-resistant *Staphylococcus aureus*; and VRE, vancomycin-resistant enterococci.

We used data reported to the Hospital Compare program from the National Healthcare Safety Network for 1682 hospitals for MRSA bacteremia, 2293 hospitals for CAUTI, 2029 hospitals for CLABSI, and 3055 hospitals for CDI. We used data from 20 ICUs across the United States for MRSA and VRE acquisition. The Table demonstrates the total number of observed cases, total patient-days or device-days, and daily estimated rates for HAI, MRSA, and VRE acquisition. Cluster-specific calculated mean daily rate of infection (or acquisition), SD, and CV for each outcome are also shown in the Table. Figure 1 also graphically represents the variation across our outcomes’ cluster-specific rates.

### MRSA Bacteremia

**Figure 2.**
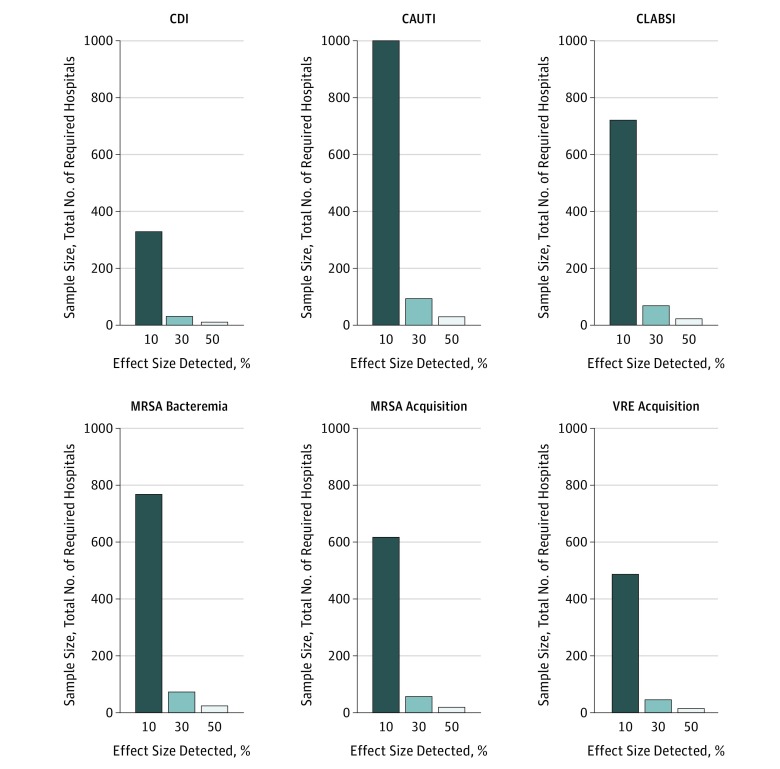
Association of Intervention Effect Size With Sample Size Requirements by Outcome With Coefficient of Variation Held Constant Sample sizes refer to total number of clusters based on a 1-year, 2-group parallel cluster-randomized trial. Mean cluster sizes vary. CAUTI indicates catheter-associated urinary tract infections; CDI, *Clostridium difficile* infection; CLABSI, central-line–associated bloodstream infections; MRSA, methicillin-resistant *Staphylococcus aureus*; and VRE, vancomycin-resistant enterococci.

**Figure 3.**
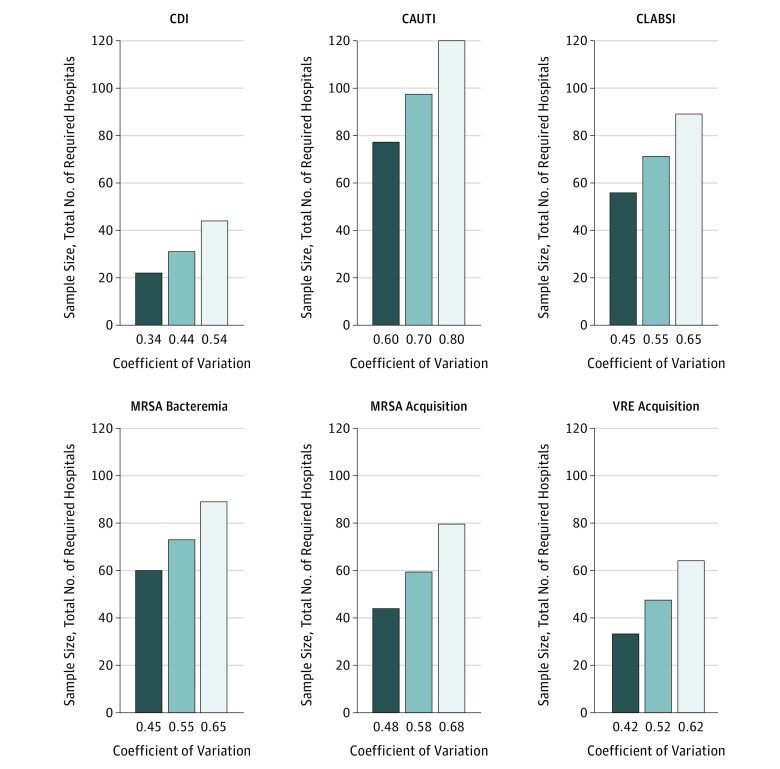
Association of Coefficient of Variation With Sample Size Requirements by Outcome With Effect Size Held Constant at 30% Sample sizes refer to total number of clusters based on a 1-year, 2-group parallel cluster-randomized trial. Mean clusters sizes vary. CAUTI indicates catheter-associated urinary tract infections; CDI, *Clostridium difficile* infection; CLABSI, central-line–associated bloodstream infections; MRSA, methicillin-resistant *Staphylococcus aureus*; and VRE, vancomycin-resistant enterococci.

Figure 2 and Figure 3 illustrate the estimated total number of clusters needed to optimally power a parallel CRT for each HAI and acquisition outcome using different assumptions. For MRSA bacteremia, if researchers were studying an intervention such as MRSA decolonization and thought that the intervention would lead to a 30% decrease in the daily rate of MRSA bacteremia, using a CV of 0.55 (calculated from the National Healthcare Safety Network data) and assuming a mean of 219 patients per day per cluster, one would need a total of 73 clusters (37 in the intervention group and 36 in the control group) to observe a statistically significant decrease, assuming a 1-year study with a type I error rate of 0.05 and a type II error rate of 0.20 (power of 0.80). If the expected effect size decreased to 10%, one would need a total of 768 clusters. If instead the CV were 0.45, one would need a total of 60 clusters to observe a 30% decrease in MRSA bacteremia daily rate or 626 clusters to observe a 10% decrease.

### Catheter-Associated Urinary Tract Infections

For CAUTI, using a CV of 0.70 and assuming a mean of 23 patients at risk for CAUTI per day per cluster, 82 clusters in total (41 in the intervention group and 41 in the control group) are required to observe a 30% decrease in CAUTI daily rate, assuming a 1-year study with a type I error rate of 0.05 and a type II error rate of 0.20. If the anticipated effectiveness of the intervention were 10%, 875 clusters would be needed. If instead the CV were 0.60, one would need a total of 65 clusters to observe a 30% decrease in CAUTI daily rate or 690 clusters to observe a 10% decrease.

### Central-Line–Associated Bloodstream Infections

For CLABSI, using the calculated CV of 0.55 and assuming a mean of 25 patients at risk for CLABSI per day per cluster, 60 clusters in total (30 in the intervention group and 30 in the control group) are required to observe a 30% decrease in CLABSI daily rate, assuming a 1-year study with a type I error rate of 0.05 and a type II error rate of 0.20. If the effectiveness of the intervention were 10%, 631 clusters would be needed. If instead the CV were 0.45, one would need a total of 47 clusters to observe a 30% decrease in CLABSI daily rate or 489 clusters to observe a 10% decrease.

### *Clostridium difficile* Infections

For CDI, using the calculated CV of 0.44 and assuming a mean of 124 patients at risk for CDI per day per cluster, 31 clusters in total (16 in the intervention group and 15 in the control group) are required to observe a 30% decrease in CDI daily rate, assuming a 1-year study with a type I error rate of 0.05 and a type II error rate of 0.20. If the effectiveness of the intervention were 10%, 329 clusters would be needed. If the CV were 0.34, a total of 22 clusters would be required to observe a 30% decrease in CDI daily rate or 218 clusters to observe a 10% decrease. For all HAIs evaluated, changing power from 0.80 to 0.90 did not change the sample calculations considerably (eTable 1 in the Supplement).

### MRSA and VRE Acquisition

For MRSA acquisition, using the calculated CV of 0.58 and assuming a mean of 7 patients at risk for MRSA acquisition per day per cluster, 50 clusters in total (25 in the intervention group and 25 in the control group) are required to observe a 30% decrease in MRSA acquisition daily rate, assuming a 1-year study with a type I error rate of 0.05 and a type II error rate of 0.20. If the effectiveness of the intervention were 10%, 540 clusters would be needed. If instead the CV were 0.48, a total of 37 clusters would be necessary to observe a 30% decrease in MRSA acquisition daily rate or 389 clusters to observe a 10% decrease. For VRE acquisition, using the calculated CV of 0.52 and assuming a mean of 7 patients at risk for VRE acquisition per day per cluster, 40 clusters are required to observe a 30% decrease in VRE acquisition daily rate, assuming a 1-year study with a type I error rate of 0.05 and a type II error rate of 0.20. If the effectiveness of the intervention were 10%, 426 clusters would be needed. If instead the CV were 0.42, one would need a total of 28 clusters to observe a 30% decrease in VRE acquisition daily rate or 292 clusters to observe a 10% decrease. As demonstrated with the HAI outcomes, changing power from 0.80 to 0.90 also did not change the sample calculations considerably for MRSA and VRE acquisition outcomes (eTable 2 in the Supplement).

## Discussion

Our results demonstrate the large sample sizes needed to adequately power parallel CRTs with the most commonly used hospital epidemiology and antimicrobial stewardship outcomes. Furthermore, the estimated sample sizes were larger than conventionally thought. Our findings also show that sample size calculations were most strongly associated with the effectiveness of the intervention and the intraclass correlation or lack of independence of patients within a cluster. The sample sizes presented herein are intended to illustrate what might be necessary in practice. Other parameters used in the calculation (such as the planned follow-up time and allocation ratio) may vary from study to study, and we therefore encourage investigators to use the methods presented herein only as a guide while calculating their own sample size estimates.

These findings support the following 4 main points. First, valid and reliable cluster trials in the field of hospital epidemiology are going to be costly. Second, the large effect of correlation on the results demonstrates the critical importance of understanding the ICC and CV in the study population before embarking on this type of study. Published CRTs often do not report their ICC or CV, hindering future researchers from making informed decisions when designing new CRTs.^20,21^ Journals should consider implementing guidelines to normalize the practice of reporting such measures. Third, the expected effect size of the intervention has an important association with sample size. Although we presented a range of effect sizes, observing a 50% decrease in outcome is extremely uncommon in this field of study. However, recently published CRTs were powered expecting large effect sizes (ie, 40%-60%).^7,22^ Non–statistically significant results observed in these studies are difficult to interpret because they may be underpowered and unable to statistically detect a smaller effect size. Finally, acquisition outcomes (ie, MRSA and VRE acquisition) are more common than HAI outcomes; therefore, as expected, using acquisition outcomes instead of HAI outcomes reduces the sample size requirements to perform appropriately powered studies. Preliminary data should be collected to better estimate effect size, ICC, and CV.

### Limitations

This study presents sample size estimates for simple parallel CRTs with only 1 level of clustering and does not account for rates in each hospital at baseline. Also, clustering may arise at more than 1 level. For example, the variation among hospitals and the variation among ICUs within hospitals present 2 levels of clustering. In this situation, it would be necessary to use 2 ICCs in the sample size calculations. Similarly, the approach would also differ slightly for matched, stratified, or cluster crossover designs. We estimated the SD of the CVs (Table) and found that estimates were very precise owing to the large sample size. The SD was not used to change the CV parameter. Instead, we chose to increase and decrease the CV by 0.1 to illustrate the effect of the value of the CV instead of the precision of the estimate. We note that the variance was greater for the acquisition outcomes; however, for consistency, we used the same approach. In addition, we did not include in this study a description of the hospitals that constituted our data set; however, the incidence of infection and CV could vary by hospital attributes (eg, hospital size, academic affiliation, urban/rural location) used by investigators to select study facilities. Also, as with most studies, we did not account for the effect of cluster size variation, despite the data suggesting that this variability can lead to a decrease in power.^20^

A major strength of our study was that CV estimates for infection outcomes were derived from publicly available Hospital Compare data and therefore represent most US hospitals participating in the Centers for Medicare & Medicaid Services Inpatient and Prospective Payment System. However, the estimates made for acquisition outcomes (ie, MRSA, VRE) may not be as generalizable because they were based on only 1 study, although the BUGG study was the largest measuring colonization acquisition and included 20 different ICUs across the United States.

## Conclusions

There is a growing call for fewer, higher-quality, more conclusive trials in the field of hospital epidemiology. We hope that the findings presented herein lead to more carefully designed, definitive, controlled CRTs that are properly powered and that more studies report the parameters used to generate their sample size estimates.

## Notes

Supplement. **eTable 1.** Sample Size Parameters and Estimations for Health Care–Associated Infection (HAI) Parallel Cluster Randomized Trials **eTable 2.** Sample Size Parameters and Estimations for MRSA and VRE Acquisition Parallel Cluster Randomized Trials **eMethods.** Equation to Estimate the Variance of Site-Specific Rates Given Data on Observed Rates at Multiple Sites
