# Supplementary material for: Sample Size Estimates for Cluster-Randomized Trials in Hospital Infection Control and Antimicrobial Stewardship
Source: JAMA Netw Open. 2019 Oct 4;2(10):e1912644. doi: 10.1001/jamanetworkopen.2019.12644 (PMC6784749; doi:10.1001/jamanetworkopen.2019.12644)
Supplement: Supplement. — eTable 1. Sample Size Parameters and Estimations for Health Care–Associated Infection (HAI) Parallel Cluster Randomized Trials eTable 2. Sample Size Parameters and Estimations for MRSA and VRE Acquisition Parallel Cluster Randomized Trials eMethods. Equation to Estimate the Variance of Site-Specific Rates Given Data on Observed Rates at Multiple Sites [file jamanetwopen-2-e1912644-s001.pdf]

Supplementary Online Content

Blanco N, Harris AD, Magder LS, et al. Sample size estimates for cluster-randomized trials in hospital infection control and antimicrobial stewardship. *JAMA Netw Open.* 2019;2(10):e1912644. doi:10.1001/jamanetworkopen.2019.12644

eTable 1. Sample Size Parameters and Estimations for Health Care–Associated Infection (HAI) Parallel Cluster Randomized Trials

eTable 2. Sample Size Parameters and Estimations for MRSA and VRE Acquisition Parallel Cluster Randomized Trials

eMethods. Equation to Estimate the Variance of Site-Specific Rates Given Data on Observed Rates at Multiple Sites

This supplementary material has been provided by the authors to give readers additional information about their work.

© 2019 Blanco N et al. *JAMA Network Open*

eTable 1. Sample Size Parameters and Estimations for Health Care–Associated Infection (HAI) Parallel Cluster Randomized Trials

**MRSA Bacteremia**												
Anticipated incidence rate (intervention) (effect size)	(50%) 0.0000275	(30%) 0.0000385	(10%) 0.0000495	(50%) 0.0000275	(30%) 0.0000385	(10%) 0.0000495	(50%) 0.0000275	(30%) 0.0000385	(10%) 0.0000495	(50%) 0.0000275	(30%) 0.0000385	(10%) 0.0000495
Anticipated incidence rate (control)	0.000055	0.000055	0.000055	0.000055	0.000055	0.000055	0.000055	0.000055	0.000055	0.000055	0.000055	0.000055
Cluster size	219.4	219.4	219.4	219.4	219.4	219.4	219.4	219.4	219.4	219.4	219.4	219.4
CV	0.45	0.45	0.45	**0.55**	**0.55**	**0.55**	0.65	0.65	0.65	**0.55**	**0.55**	**0.55**
Power	0.8	0.8	0.8	0.8	0.8	0.8	0.8	0.8	0.8	0.9	0.9	0.9
**TOTAL SAMPLE SIZE (hospitals)**	**20**	**60**	**626**	**24**	**73**	**768**	**29**	**89**	**938**	**31**	**98**	**1028**
**CAUTI**												
Anticipated incidence rate (intervention) (effect size)	(50%) 0.0004775	(30%) 0.0006685	(10%) 0.0008595	(50%) 0.0004775	(30%) 0.0006685	(10%) 0.0008595	(50%) 0.0004775	(30%) 0.0006685	(10%) 0.0008595	(50%) 0.0004775	(30%) 0.0006685	(10%) 0.0008595
Anticipated incidence rate (control)	0.000955	0.000955	0.000955	0.000955	0.000955	0.000955	0.000955	0.000955	0.000955	0.000955	0.000955	0.000955
Cluster size	23.3	23.3	23.3	23.3	23.3	23.3	23.3	23.3	23.3	23.3	23.3	23.3
CV	0.60	0.60	0.60	**0.70**	**0.70**	**0.70**	0.80	0.80	0.80	**0.70**	**0.70**	**0.70**
Power	0.8	0.8	0.8	0.8	0.8	0.8	0.8	0.8	0.8	0.9	0.9	0.9
**TOTAL SAMPLE SIZE (hospitals)**	**21**	**65**	**690**	**26**	**82**	**875**	**31**	**101**	**1088**	**34**	**109**	**1171**
**CLABSI**												

© 2019 Blanco N et al. *JAMA Network Open*

Anticipated incidence rate (intervention) (effect size)	(50%) 0.0004075	(30%) 0.0005705	(10%) 0.0007335	(50%) 0.0004075	(30%) 0.0005705	(10%) 0.0007335	(50%) 0.0004075	(30%) 0.0005705	(10%) 0.0007335	(50%) 0.0004075	(30%) 0.0005705	(10%) 0.0007335
Anticipated incidence rate (control)	0.000815	0.000815	0.000815	0.000815	0.000815	0.000815	0.000815	0.000815	0.000815	0.000815	0.000815	0.000815
Cluster size	25.0	25.0	25.0	25.0	25.0	25.0	25.0	25.0	25.0	25.0	25.0	25.0
CV	0.45	0.45	0.45	**0.55**	**0.55**	**0.55**	0.65	0.65	0.65	**0.55**	**0.55**	**0.55**
Power	0.8	0.8	0.8	0.8	0.8	0.8	0.8	0.8	0.8	0.9	0.9	0.9
**TOTAL SAMPLE SIZE (hospitals)**	**16**	**47**	**489**	**20**	**60**	**631**	**24**	**75**	**801**	**26**	**80**	**844**
**C. *difficile* Infection**												
Anticipated incidence rate (intervention) (effect size)	(50%) 0.0003080	(30%) 0.0004312	(10%) 0.0005544	(50%) 0.0003080	(30%) 0.0004312	(10%) 0.0005544	(50%) 0.0003080	(30%) 0.0004312	(10%) 0.0005544	(50%) 0.0003080	(30%) 0.0004312	(10%) 0.0005544
Anticipated incidence rate (control)	0.000616	0.000616	0.000616	0.000616	0.000616	0.000616	0.000616	0.000616	0.000616	0.000616	0.000616	0.000616
Cluster size	124.2	124.2	124.2	124.2	124.2	124.2	124.2	124.2	124.2	124.2	124.2	124.2
CV	0.34	0.34	0.34	**0.44**	**0.44**	**0.44**	0.54	0.54	0.54	**0.44**	**0.44**	**0.44**
Power	0.8	0.8	0.8	0.8	0.8	0.8	0.8	0.8	0.8	0.9	0.9	0.9
**TOTAL SAMPLE SIZE (hospitals)**	**8**	**22**	**218**	**11**	**31**	**329**	**15**	**44**	**468**	**14**	**41**	**440**

Anticipated incidence rate (intervention) (Ie) = The anticipated daily average incidence rate in the experimental group with the outcome.

Anticipated incidence rate (control) (Ic) = The anticipated daily average incidence rate in the control group with the outcome.

Effect size= % difference between the anticipated daily average incidence rate in the experimental group with the outcome and the anticipated daily average incidence rate in the control group with the outcome

Cluster size (m)= The anticipated average (or actual) cluster size

CV = The coefficient of variation of cluster-specific incidence rates, assumed constant over both the treatment and control groups. The bolded CV was estimated from Hospital Compare data.

Power = The desired level of power, recall power = 1 - type II error

Other parameters included (and held constant) but not shown in table:

Planned follow-up time (t) for the study (days)= 365

© 2019 Blanco N et al. *JAMA Network Open*

Allocation Ratio (AR) of patients in intervention group to control group=1
Alpha or the desired type I error rate= 0.05

© 2019 Blanco N et al. *JAMA Network Open*

eTable 2. Sample Size Parameters and Estimations for MRSA and VRE Acquisition Parallel Cluster Randomized Trials

**MRSA Acquisition**												
Anticipated incidence rate (intervention) (effect size)	(50%) 0.004160	(30%) 0.005824	(10%) 0.007488	(50%) 0.004160	(30%) 0.005824	(10%) 0.007488	(50%) 0.004160	(30%) 0.005824	(10%) 0.007488	(50%) 0.004160	(30%) 0.005824	(10%) 0.007488
Anticipated incidence rate (control)	0.008320	0.008320	0.008320	0.008320	0.008320	0.008320	0.008320	0.008320	0.008320	0.008320	0.008320	0.008320
Cluster size	7.1	7.1	7.1	7.1	7.1	7.1	7.1	7.1	7.1	7.1	7.1	7.1
CV	0.48	0.48	0.48	**0.58**	**0.58**	**0.58**	0.68	0.68	0.68	**0.58**	**0.58**	**0.58**
Power	0.8	0.8	0.8	0.8	0.8	0.8	0.8	0.8	0.8	0.9	0.9	0.9
**TOTAL SAMPLE SIZE (hospitals)**	**13**	**37**	**389**	**17**	**50**	**540**	**22**	**67**	**719**	**22**	**67**	**722**
**VRE Acquisition**												
Anticipated incidence rate (intervention) (effect size)	(50%) 0.007130	(30%) 0.00998	(10%) 0.012834	(50%) 0.007130	(30%) 0.00998	(10%) 0.012834	(50%) 0.007130	(30%) 0.00998	(10%) 0.012834	(50%) 0.007130	(30%) 0.00998	(10%) 0.012834
Anticipated incidence rate (control)	0.014260	0.014260	0.014260	0.014260	0.014260	0.014260	0.014260	0.014260	0.014260	0.014260	0.014260	0.014260
Cluster size	6.9	6.9	6.9	6.9	6.9	6.9	6.9	6.9	6.9	6.9	6.9	6.9
CV	0.42	0.42	0.42	**0.52**	**0.52**	**0.52**	0.62	0.62	0.62	**0.52**	**0.52**	**0.52**
Power	0.8	0.8	0.8	0.8	0.8	0.8	0.8	0.8	0.8	0.9	0.9	0.9
**TOTAL SAMPLE SIZE (hospitals)**	**10**	**28**	**292**	**13**	**40**	**426**	**18**	**54**	**588**	**17**	**53**	**570**

Anticipated incidence rate (intervention) (Ie) = The anticipated daily average incidence rate in the experimental group with the outcome.
Anticipated incidence rate (control) (Ic) = The anticipated daily average incidence rate in the control group with the outcome.
Effect size= % difference between the anticipated daily average incidence rate in the experimental group with the outcome and the anticipated daily average incidence rate in the control group with the outcome
Cluster size (m)= The anticipated average (or actual) cluster size
CV = The coefficient of variation of cluster-specific incidence rates, assumed constant over both the treatment and control groups. The bolded CV was estimated from Hospital Compare data.
Power = The desired level of power, recall power = 1 - type II error
Other parameters included (and held constant) but not shown in table:

© 2019 Blanco N et al. *JAMA Network Open*

Planned follow-up time (t) for the study (days)= 120
Allocation Ratio (AR) of patients in intervention group to control group=1
Alpha or the desired type I error rate= 0.05=

© 2019 Blanco N et al. *JAMA Network Open*

eMethods. Equation to Estimate the Variance of Site-Specific Rates Given Data on Observed Rates at Multiple Sites

Let Yi and Ti stand for the number of acquired infections, and the total patient-time observed in cluster "i".

Assume:

$Yi \mid \lambda_i \sim \text{Poisson}(\lambda_i, Ti)$ where λ_i is the site-specific rate of acquired infections.

Let σ^2_λ stand for the variance of the site-specific rates. This is the parameter of interest.

Define $\hat{\lambda}_i$ = Yi/Ti

Now

$$\begin{aligned} \text{Var}(\hat{\lambda}_i) &= \text{E}(\text{Var}(\hat{\lambda}_i \mid \lambda i)) + \text{Var}(\text{E}(\hat{\lambda}_i \mid \lambda i) \\ &= \text{E}(\lambda i/Ti) + \text{Var}(\lambda i) \\ &= \lambda/Ti + \sigma^2_\lambda \end{aligned}$$

Now suppose we observe a set of independent Yi, i=1 to n, and we want to estimate σ^2_λ and λ

However, let AVEvar=Average (Var($\hat{\lambda}_i$)) which equals:

$$\text{AVEvar} = \frac{1}{n}\sum\left((\lambda/T_i) + \sigma_\lambda^2\right) = \frac{1}{n}\lambda\sum(1/T_i) + \sigma_\lambda^2$$

Which implies that

$$\sigma^2_\lambda = \text{AVEvar} - \frac{1}{n}\lambda\sum 1/Ti \quad (1)$$

Note, we can get a natural estimate of the AVEvar using

$$\sum_{i=1}^{n}(\hat{\lambda}_i - \lambda)^2$$

where λ is estimated as the mean or a weighted mean of the $\hat{\lambda}_i$.

Plugging that estimate for AVEvar into equation (1) gives us an estimate of σ^2_λ .

© 2019 Blanco N et al. *JAMA Network Open*
